# The Feasibility and Oncological Safety of Axillary Reverse Mapping in Patients with Breast Cancer: A Systematic Review and Meta-Analysis of Prospective Studies

**DOI:** 10.1371/journal.pone.0150285

**Published:** 2016-02-26

**Authors:** Chao Han, Ben Yang, Wen-Shu Zuo, Gang Zheng, Li Yang, Mei-Zhu Zheng

**Affiliations:** 1 Department of Surgery II, Breast Cancer Center, Shandong Cancer Hospital and Institute, Jinan, Shandong, China; 2 School of Medicine and life Sciences, University of Jinan-Shandong Academy of Medical Sciences, Jinan, Shandong, China; The First Affiliated Hospital with Nanjing Medical University, CHINA

## Abstract

**Objective:**

The axillary reverse mapping (ARM) technique has recently been developed to prevent lymphedema by preserving the arm lymphatic drainage during sentinel lymph node biopsy (SLNB) or axillary lymph node dissection (ALND) procedures. The objective of this systematic review and meta-analysis was to evaluate the feasibility and oncological safety of ARM.

**Methods:**

We searched Medline, Embase, Web of science, Scopus, and the Cochrane Library for relevant prospective studies. The identification rate of ARM nodes, the crossover rate of SLN-ARM nodes, the proportion of metastatic ARM nodes, and the incidence of complications were pooled into meta-analyses by the random-effects model.

**Results:**

A total of 24 prospective studies were included into meta-analyses, of which 11 studies reported ARM during SLNB, and 18 studies reported ARM during SLNB. The overall identification rate of ARM nodes was 38.2% (95% CI 32.9%-43.8%) during SLNB and 82.8% (78.0%-86.6%) during ALND, respectively. The crossover rate of SLN-ARM nodes was 19.6% (95% CI 14.4%-26.1%). The metastatic rate of ARM nodes was 16.9% (95% CI 14.2%-20.1%). The pooled incidence of lymphedema was 4.1% (95% CI 2.9–5.9%) for patients undergoing ARM procedure.

**Conclusions:**

The ARM procedure was feasible during ALND. Nevertheless, it was restricted by low identification rate of ARM nodes during SLNB. ARM was beneficial for preventing lymphedema. However, this technique should be performed with caution given the possibility of crossover SLN-ARM nodes and metastatic ARM nodes. ARM appeared to be unsuitable for patients with clinically positive breast cancer due to oncological safety concern.

## Introduction

Breast cancer is the most common malignancy among women in the United States and is the second leading cause of cancer-related death [1, 2]. The status of axillary lymph nodes is one of the most important prognostic factors for patients with breast cancer, and can directly guide adjuvant therapy choices [1]. Currently, axillary lymph node dissection (ALND) represents the standard treatment for patients with metastatic axillary lymph nodes [1]. However, ALND always carries an unacceptable high incidence of lymphedema, ranging from 6% to 57% [3]. For patients with clinically negative axilla, Sentinel lymph nodes biopsy (SLNB) is recommended for the surgical staging, with significantly decreased surgical complications compared with ALND [1, 4]. Nevertheless, the incidence of lymphedema remains significant, ranging from 0 to 13% [5].

Since 2007, axillary reverse mapping (ARM) has been developed as a novel surgical approach to distinguish the lymphatic drainage pattern of the upper limb from that of the breast [6, 7]. It could be performed accompanying with ALND or SLNB procedures. The successful identification and preservation of ARM nodes/lymphatics are prerequisites for ARM feasibility. However, the identification rates of lymphatics or nodes during ARM varied between previous studies [8, 9]. As the converged ARM-SLN nodes were unlikely to be preserved during sentinel node biopsy, their proportion was also closely related to ARM feasibility [10, 11]. In addition, the preserved ARM nodes should not contain metastasis. The metastatic rate of ARM nodes during ALND could reflect the oncological safety of ARM. So far, no published guideline has appraised the role of ARM in breast cancer [1, 12]. Therefore, we carried out this systematic review and meta-analysis, aiming to assess the feasibility and oncological safety of ARM during SLNB or ALND procedures.

## Method

### Search Strategy

This systematic review and meta-analysis was conducted according to guidelines from the Preferred Reporting Items for Systematic Reviews and Meta-Analyses (PRISMA) [13]. We searched Medline (Ovid format), Embase, Web of science, Scopus, and Cochrane Library were searched from their inception until September 2015. We used the following Mesh Terms or key words in the search: “axillary reverse mapping”, “lymphatic arm drainage”, and “breast cancer”. The search strategy was shown in S1 Table. The language was restricted to English. The references included articles were manually searched for additional relevant records.

### Inclusion Criteria

Studies were considered to be eligible if they met the following criteria: (i) including patients with breast cancer who underwent ARM procedures during SLNB and/or ALND; (ii) full-text articles published in English; (iii) prospectively designed, being randomized controlled trials (RCTs) and prospective non-randomized studies; (iv) reporting data on outcomes of interest. With respect to ARM procedures, the feasibility lied in sufficient identification of lymph nodes and/or lymphatics. The oncological safety was mainly represented by a low rate of positive resect ARM nodes, and a low rate of converged SLN-ARM nodes. Thus, the primary outcomes were defined as the overall identification rate of lymph nodes and/or lymphatics, the rate of positive resected ARM nodes, and the rate of converged SLN-ARM nodes. The second outcome was the incidence of lymphedema during follow-up. The occurrences of lymphedema measured within 3 months of ARM procedure were excluded because arm-related changes during this timeframe potentially represented acute surgery-related response [14]. In addition, we tried to assess the influences of preoperative neoadjuvant chemotherapy and axillary metastasis on the metastasis rate of ARM nodes. The staging of breast cancer was defined according to the 2015 NCCN guideline [1]. We compared the results between pN_0-1_ and pN_2-3_ stages of breast cancer.

### Data Extraction and Quality Assessment

Two authors (CH and BY) independently extracted all data, with discrepancies resolved by consensus or discussion with a third investigator (WSZ). The following data were extracted: author, publication year, location, number of patients, mean/median age, mapping material for ARM, number of ARM procedures during SLNB and/or ALND, outcomes, and study period. Data on ARM during SLNB or ALND procedures were extracted separately. The quality of included studies was assessed by using the Agency for Healthcare Research and Quality (AHRQ) checklist [15].

### Statistical Analysis

The event rates for outcomes of interest were combined to determine the pooled rates and accompanying 95% confidence intervals (CIs). The Comprehensive Meta-Analysis statistical package (CMA Version 2.2, Biostat, Englewood, NJ) was used to conduct all meta-analyses by employing random-effects models. The heterogeneity across the results of included studies was assessed by using I^2^ statistics and the χ^2^-test. Low, moderate and high heterogeneity was set at I^2^ values of 25%, 50% and 75%, respectively [16]. We did separate analyses for ARM-SLNB procedures and ARM-ALND procedures. Subgroup analyses were performed according to different ARM mapping materials (blue dye, isotope, or fluorescence) and different locations (Asia, Europe, or North America). Meta-regression analyses (unrestricted maximum likelihood) were performed to determine whether the pooled rates were modulated by sample sizes. The publication bias was inspected visually by the funnel plots and statistically by the Egger’s test [17, 18]. A P value of less than 0.1 was considered statistically significant when assessing heterogeneity or publication bias. In other ways, a P value of 0.05 was regarded as significance level.

## Results

### Study Selection

**Fig 1:**
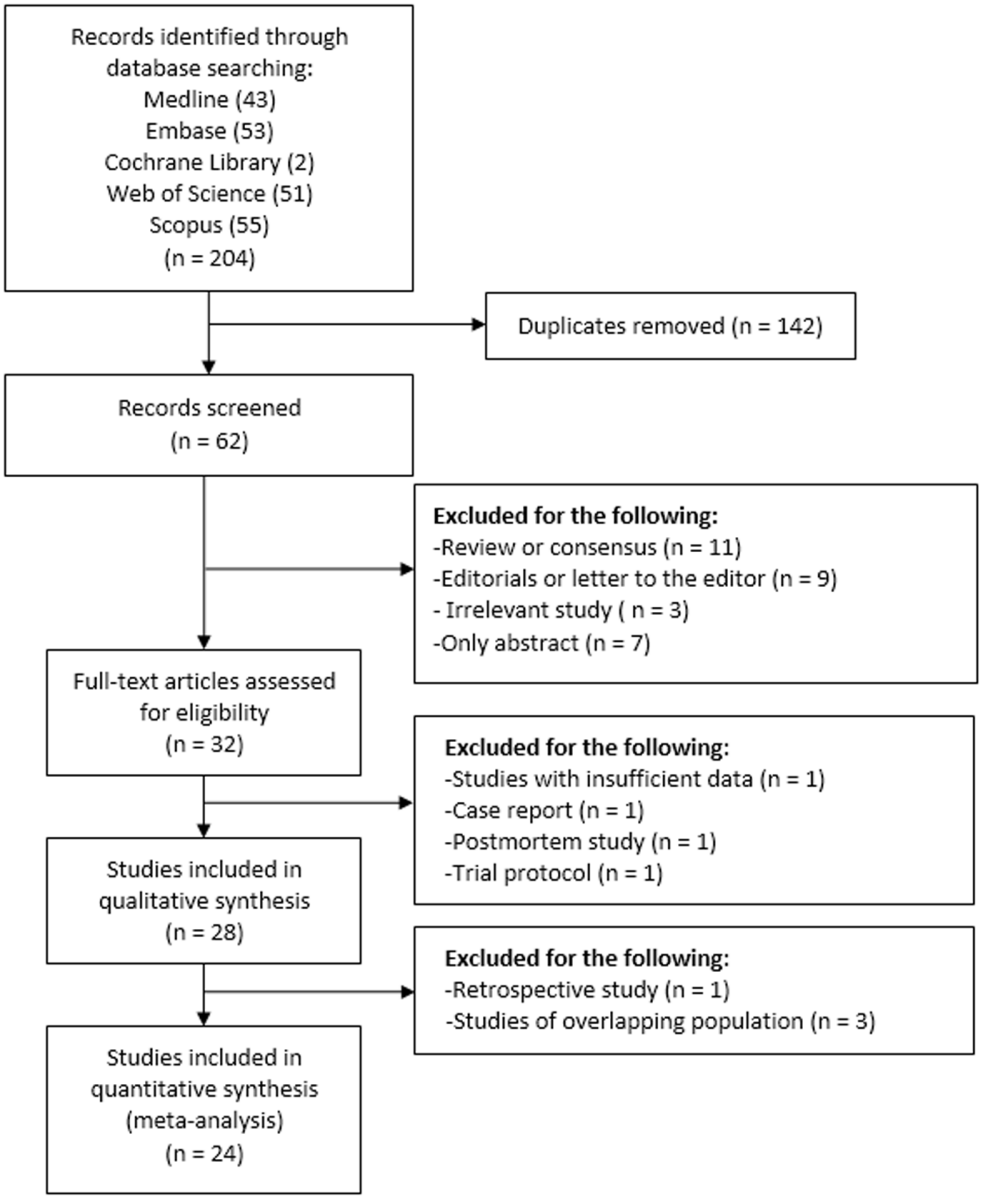
The selection process of included studies.

Our initial searches identified 95 publications, including 43 records in Medline, 53 records in Embase, 2 records in the Cochrane Library, 51 records in the Web of Science, and 55 records in the Scopus. After removing 142 duplicates, we screened 62 publications by titles and abstracts. Thirty-two records were eligible for full-text assessment. Further, one trial protocol [19], one postmortem study [20], and one case report were excluded [21]. One study were discarded because the outcomes of interest were not reported [22]. Twenty-eight studies were included into qualitative synthesis. Subsequently, one retrospective study and three studies with overlapping population were discarded [3, 10, 23, 24]. The remaining 24 publications were pooled into meta-analysis, involving 2709 patients [5–9, 11, 25–42]. (Fig 1)

### Characteristics of Included Studies

Eleven studies performed ARM procedures during SLNB [5, 9, 11, 27, 28, 30–32, 37–39], and 20 studies performed ARM procedures during ALND [6–8, 11, 25, 26, 28–38, 40–42]. All studies were prospectively designed, with 23 singly-arm studies performing ARM during SLNB or ALND, and only 1 randomized controlled trial comparing the outcomes between ARM patients and non-ARM patients [42]. With respect to ARM mapping materials, 17 studies used blue dye alone [5–7, 9, 11, 25–30, 32, 36–38, 40, 41]; 2 studies used fluorescence alone [31, 35]; 1 study used blue dye in combination with fluorescence [39]; 3 studies used blue dye together with radioisotope [8, 34, 42]; and 1 study used radioisotope [33]. Seven studies were from North America [5, 7, 11, 26, 28, 37, 38], nine from Europe [6, 8, 9, 25, 29, 32–34, 41], seven from Asia [27, 30, 31, 35, 36, 39, 42], and 1 from the South America [40]. The characteristics of included studies were shown in Table 1. The included studies showed low to moderate quality, with quality scores ranging from 2 to 7 points. The items satisfied least were blindness to other aspects of the status of the participants, missing data handled in the analysis, and the patient response rates and completeness of data collection. (S2 Table)

**Table 1:** Characteristics of included studies.

Author (year)	Location	No. of Patients	Age	Procedures (n)	Mapping material	Overall identification rate of ARM nodes or lymphatics	Reported complications	Lymphedema follow-up duration	Study period
Thompson et al. (2007)	USA	40	Median: 49.7	SLNB alone (32); ALND with/without SLNB (18)	Blue dye	61.1% (11/18)	Allergic reaction; blue tattoo; lymphedema	NA	May 2006-October 2006
Nos et al. (2007)	France	21	58	ALND alone (21)	Blue dye	71.4% (15/21)	Blue tattoo	NA	November 2004-February 2005
Nos et al. (2008)	France	23	49.7	ALND alone (23)	Blue dye +radioisotope	91% (21/23)	NA	NA	July 2006-March 2008
Boneti et al. (2009)	USA	220	60.3	SLNB alone (220); ALND+SLNB (47)	Blue dye	40.6% (87/214)	Allergic reaction; blue tattoo; lymphedema	6 months	May 2006-September 2008
Casabona et al. (2009)	Italy	72	57	SLNB with or without ALND (72); ALND+SLNB (9)	Blue dye	37.5% (27/72)	Lymphedema	9 months	January 2007-July 2008
Ponzone et al. (2009)	Italy	49	NA	ALND alone (49)	Blue dye	73.5% (34/49)	Pain; allergic reaction; blue tattoo	NA	June 2007-December 2008
Bedrosian et al. (2010)	USA	30	49	ALND alone (30)	Blue dye	70% (21/30)	Blue tattoo	NA	May 2008-January 2009
Deng et al. (2011)	China	69	48	SLNB alone (69)	Blue dye	NA	Blue tattoo	NA	October 2009-August 2010
Boneti et al. (2012)	USA	148	56.9	SLNB alone (114); ALND and SLNB (42)	Blue dye	SLNB: 39% (45/114); ALND: 81% (34/42)	Lymphedema	14.6 months	May 2007-March 2010
Gobardhan et al. (2012)	Netherlands	93	Median: 56.4	ALND alone (93)	Blue dye	90.3% (84/93)	NA	NA	October 2009-June 2011
Han et al. (2012)	Korea	97	46.2	SLNB with or without ALND (97); ALND with SLNB (83)	Blue dye	SLNB: 71.4% (10/14); ALND: 84.3% (70/83)	Lymphedema	9.6 months	January 2009-October 2010
Rubio et al. (2012)	Spain	36	59.5	SLNB with ALND (15); ALND with or without SLNB (36)	Blue dye	ALND: 83.3% (30/36)	Blue tattoo	NA	July 2009-May 2010
Noguchi et al. (2012)	Japan	131	60	SLNB with or without ALND (97); ALND alone (34)	Fluorescence	ALND: 85% (32/34); SLNB: 49.5% (48/97)	Lymphedema; allergic reaction	12 months	May 2009-June 2011
Connor et al. (2013)	USA	184	60	SLNB alone (155); ALND with or without SLNB (57)	Blue dye	SLNB: 47% (73/155); ALND: 72% (41/57)	Lymphedema	12 months	December 2009-February 2012
Tausch et al. (2013)	Switzerland	143	Median: 58	ALND alone (143)	Blue dye +radioisotope	Nodes: 78% (112/143)	Lymphedema	19 months	April 2009-April 2012
Gennaro et al. (2013)	Italy	60	NA	ALND (15); selective axillary dissection (45)	Radioisotope	NA	Lymphedema	16 months	June 2009-February 2012
Ikeda et al. (2014)	Japan	76	59	ALND with or without SLNB (98)	Fluorescence	92.1% (70/76)	Lymphedema	24 months	January 2010-December 2012
Khandelwal et al. (2014)	India	51	41.4	ALND alone (51)	Blue dye	88.2% (45/51)	Blue tattoo; skin reaction	NA	May 2011-May 2013
Kuusk et al. (2014)	Canada	52	56	SLNB alone (37); ALND alone (15)	Blue dye	SLNB: 18.9% (7/37); ALND: 46.6% (7/15)	Lymphedema; blue tattoo	24 months	July 2010-November 2012
Ochoa et al. (2014)	USA	360	56	SLNB with or without ALND (348); ALND with or without SLNB (15)	Blue dye	SLNB: 33.7% (80/237); ALND: 75.4% (93/123)	Lymphedema	NA	May 2006-October 2011
Sakurai et al. (2014)	Japan	372	Median: 59	SLNB alone (321)	Blue dye +Fluorescence	32.3% (120/372)	Lymphedema	12 months	August 209-July 2012
Schunemann et al. (2014)	Brazil	45	49.4	ALND alone (45)	Blue dye	40/45	NA	NA	January 2010-October 2012
Beek et al. (2015)	Netherland	112	55.5	ALND alone (112)	Blue dye	87.5% (98/112)	NA	NA	October 2009-November 2013
Yue et al. (2015)	China	265	50.5	ALND alone (127); ALND+ARM (138)	Blue dye +radioisotope	93.5% (129/138)	Lymphedema	20 months	January 2012-March 2014

ALND, axillary lymph node dissection; ARM, axillary reverse mapping; NA, not available; SLNB, sentinel lymph node biopsy.

### ARM in SLNB Procedures

**Fig 2:**
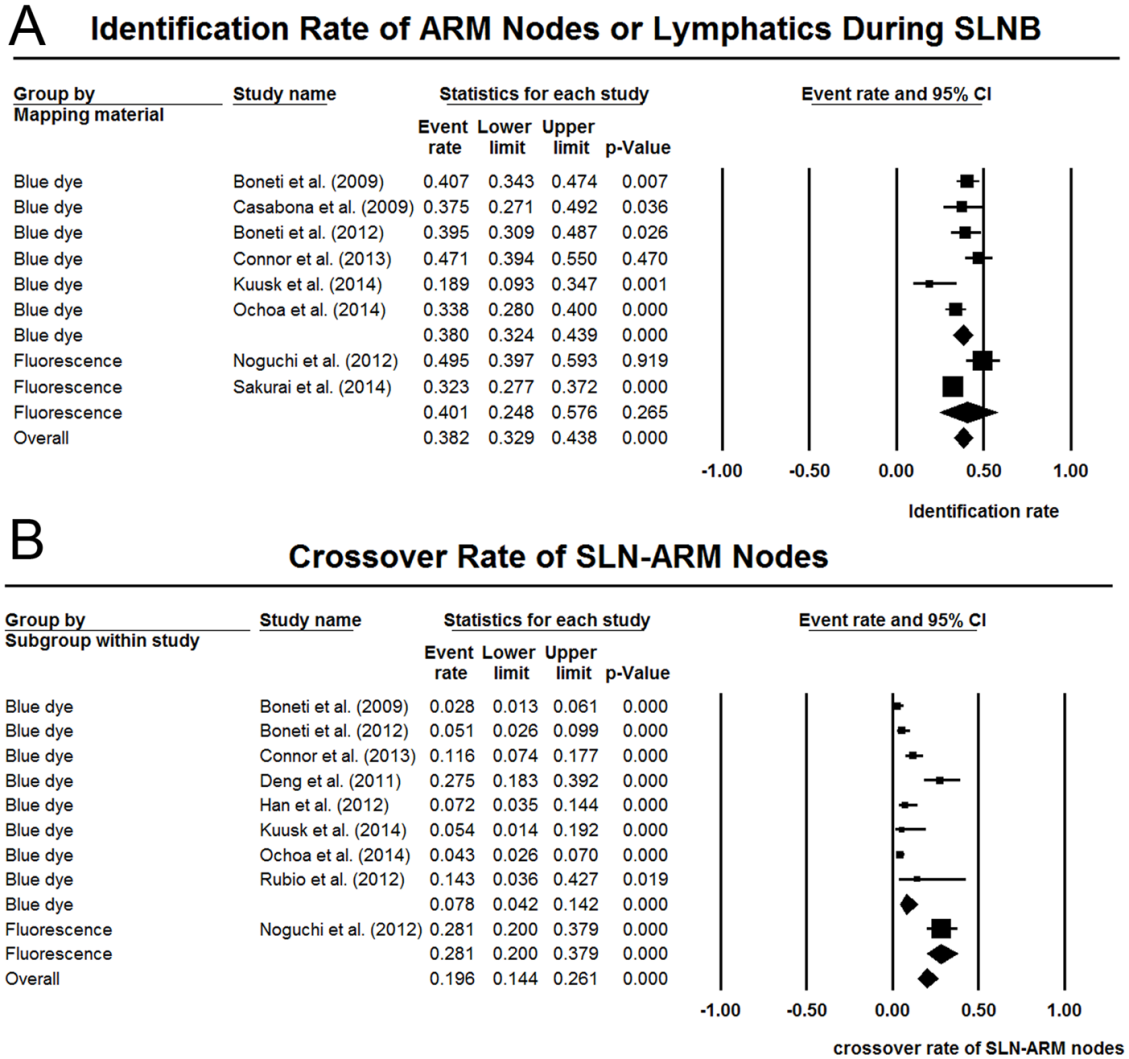
Forest plots of the pooled identification rate of ARM nodes or lymphatics, and crossover rate of ARM-SLN nodes during SLNB. (A) the identification rate of ARM nodes or lymphatics; (B) the crossover rate of ARM-SLN nodes during SLNB.

Eleven studies reported data on outcomes of ARM procedures during SLNB [5, 9, 11, 27, 28, 30–32, 37–39]. The identification rate of ARM nodes or lymphatics was reported by 8 studies [5, 9, 11, 28, 31, 37–39]. The pooled results revealed an overall identification rate of 38.2% (95% CI 32.9%-43.8%), with statistically significant heterogeneity (I^2^ = 70.5%, P < 0.05). (Fig 2A) Subgroup analyses were carried out by stratifying mapping materials and populations. The results were summarized in Table 2. Notably, the pooled identification rate remained similar in stratified analyses, with statistically significant heterogeneity across all subgroups. In meta-regression analysis, the coefficient was not statistically significant for sample size (P = 0.17). No publication bias was detected by funnel plot or the Egger’s test (P = 0.92).

**Table 2:** The results of subgroup analyses for the outcomes of identification rate and crossover rate during SLNB, respectively.

Subgroups	Number of studies	Pooled results (95% CI)	Heterogeneity (I^2^)	Heterogeneity (P)
**Identification rate**				
Overall	8	38.2% (32.9%-43.8%)	70.5%	< 0.05
Mapping material				
Blue dye	6	38.0% (32.4%-43.9%)	61.6%	< 0.05
Fluorescence	2	40.1% (24.8%-57.6%)	89.7%	< 0.05
Population				
North America	5	37.9% (31.4%-44.8%)	69.2%	< 0.05
Europe	1	37.5% (27.1%-49.2%)	-	-
Asia	2	40.1% (24.8%-57.6%)	89.7%	< 0.05
**Crossover rate**				
Overall	9	19.6% (14.4%-26.1%)	89.7%	< 0.05
Mapping material				
Blue dye	8	7.8% (4.2%-14.2%)	85.4%	< 0.05
Fluorescence	1	28.1% (20.0%-37.9%)	-	-
Population				
North America	5	5.4% (3.1%-9.4%)	71.3%	< 0.05
Europe	1	14.3% (3.6%-42.7%)	-	-
Asia	3	19.3% (9.1%-36.1%)	85.7%	< 0.05

Nine studies were available for data on the crossover rate of SLN-ARM nodes [5, 11, 27, 28, 30–32, 37, 38]. The aggregating results showed that the crossover rate of SLN-ARM nodes was 19.6% (95% CI 14.4%-26.1%), with significantly high heterogeneity (I^2^ = 89.7%, P < 0.05). (Fig 2B) When stratified by mapping materials, 8 studies of blue dye showed an overall crossover rate of 7.8% (95% CI 4.2%-14.2%), and the only study of fluorescence showed a crossover rate of 28.1% (95% CI 20.0%-37.9%) [31]. In subgroup analyses of populations, the 3 Asian studies showed a higher crossover rate than the 5 North American studies (19.3% versus 5.4%). However, the Asian result was of much wide confidential interval (95% CI 9.1%-36.1%). The pooled data were shown in Table 2. In meta-regression analysis, the coefficient was statistically significant for sample size (P = 0.03), indicating that the number of enrolled patients may modulate the crossover rate of SLN-ARM nodes.

### ARM during ALND procedures

**Fig 3:**
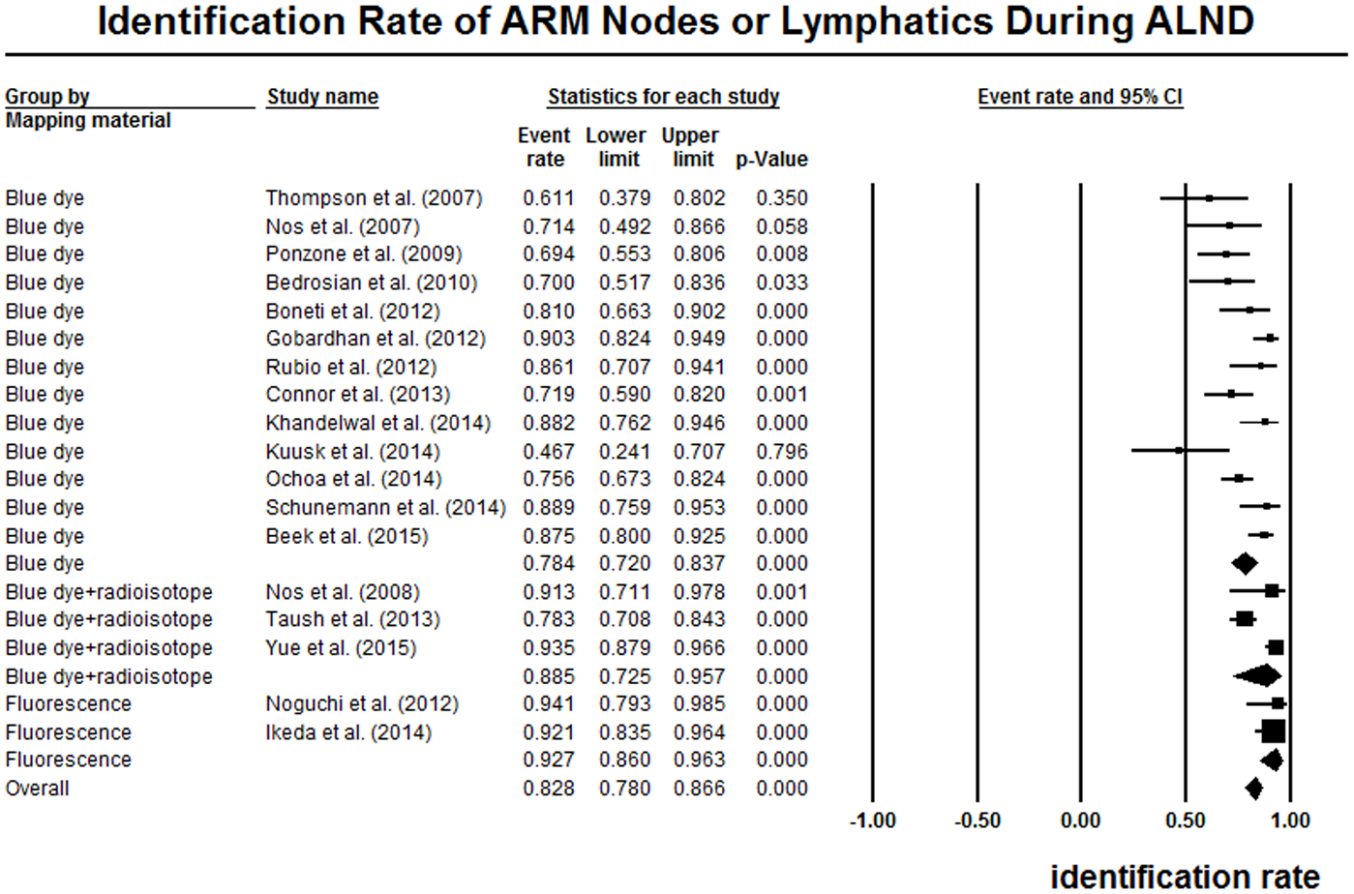
Forest plot of the pooled identification rate of ARM nodes or lymphatics during ALND.

Eighteen studies reported the identification rate of ARM nodes or lymphatics during ALND procedures [6–8, 11, 25, 26, 28, 29, 31, 32, 34–38, 40–42]. The summarized data showed an overall identification rate of 82.8% (78.0%-86.6%), with significantly high heterogeneity (I^2^ = 72.6%, P < 0.05). (Fig 3) When stratified by mapping materials, the studies of blue dye, blue dye and radioisotope, and fluorescence showed a pooled identification rate of 78.4% (95% CI 72.0%-83.7%), 88.5% (95% CI 72.5%-95.7%), and 92.7% (95% CI 86.0%-96.3%), respectively. For different populations, the North American studies, European studies, and Asian studies revealed an overall identification rate of 71.1% (95% CI 63.3%-77.8%), 82.6% (95% CI 75.5%-88.0%), and 92.1% (88.4%-94.7%), respectively. The heterogeneity remained significant in the subgroups of blue dye, blue dye combined with radioisotope, and European population. The pooled data were shown in Table 3. In meta-regression analysis, the coefficient was not statistically significant for sample size (P = 0.09). No publication bias was detected by funnel plot or the Egger’s test (P = 0.38).

**Table 3:** The results of subgroup analyses for the outcomes of identification rate and metastatic rate during ALND, respectively.

Subgroups	Number of studies	Pooled results (95% CI)	Heterogeneity (I^2^)	Heterogeneity (P)
**Identification rate**				
Overall	18	82.8% (78.0%-86.6%)	72.6%	< 0.05
Mapping material				
Blue dye	13	78.4% (72.0%-83.7%)	66.8%	< 0.05
Blue dye + radioisotope	3	88.5% (72.5%-95.7%)	84.3%	< 0.05
Fluorescence	2	92.7% (86.0%-96.3%)	0	0.71
Population				
North America	6	71.1% (63.3%-77.8%)	36.3%	0.16
Europe	7	82.6% (75.5%-88.0%)	62.5%	< 0.05
Asia	4	92.1% (88.4%-94.7%)	0	0.66
South America	1	88.9% (75.9%-95.3%)	-	-
**Metastatic rate**				
Overall	19	16.9% (14.2%-20.1%)	35.9%	0.06
Mapping material				
Blue dye	14	17.8% (14.4%-21.8%)	0	0.56
Blue dye + radioisotope	3	12.0% (8.2%-17.3%)	16.6%	0.30
Fluorescence	2	28.6% (16.2%-45.4%)	60.7%	0.11
Population				
North America	6	15.3% (8.8%-25.5%)	0	0.82
Europe	7	15.2% (12.0%-19.2%)	0	0.66
Asia	5	20.1% (15.6%-25.5%)	77%	< 0.05
South America	1	25.0% (14.0%-40.5%)	0	1.00

**Fig 4:**
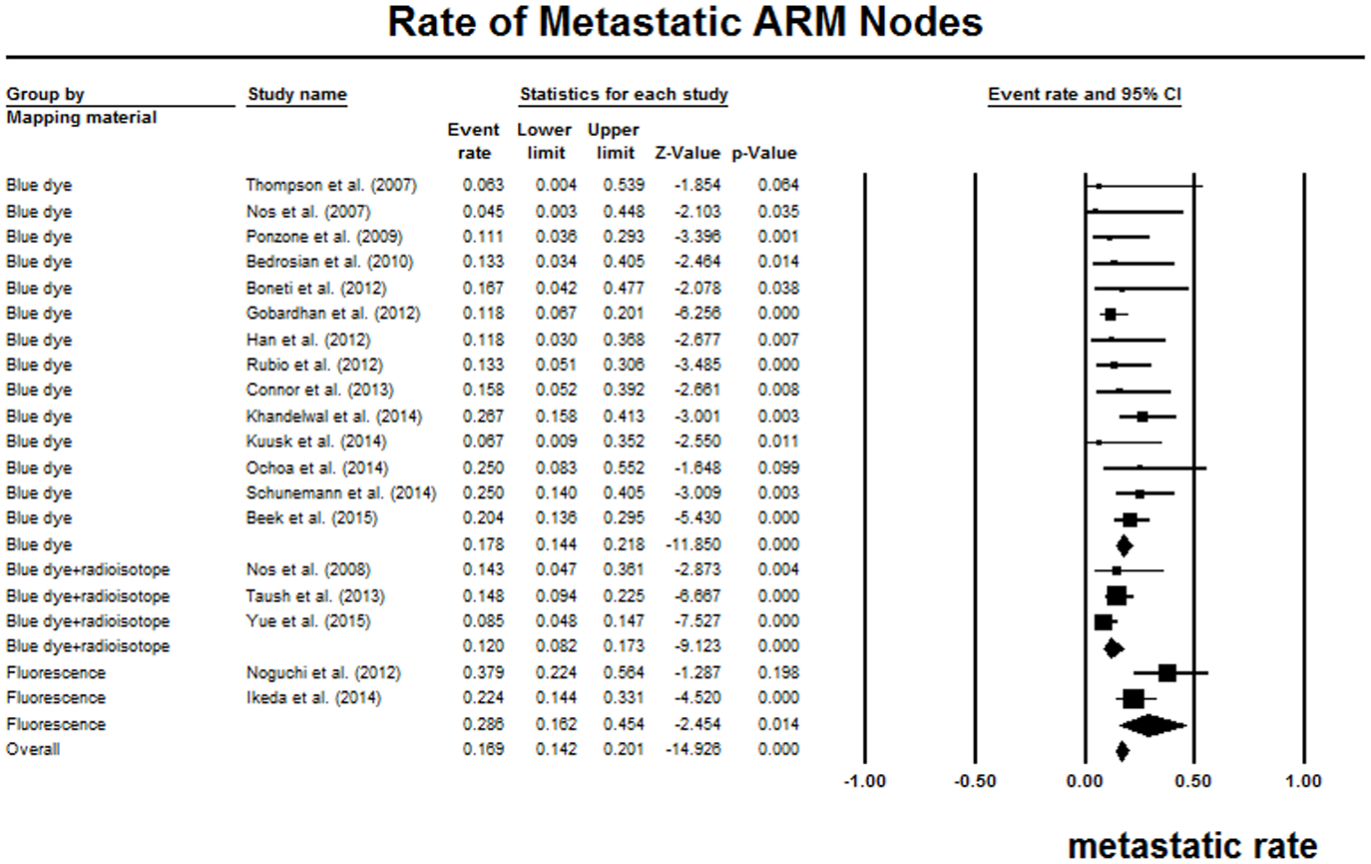
Forest plot of the pooled rate of metastasis in resected ARM nodes.

Nineteen studies reported the proportion of metastatic ARM nodes in resected ARM nodes during ALND procedures [6–8, 11, 25, 26, 28–32, 34–38, 40–42]. The pooled metastatic rate of ARM nodes was 16.9% (95% CI 14.2%-20.1%), without significant heterogeneity (I^2^ = 35.9%, P = 0.06). (Fig 4) The studies of blue dye, blue dye with radioisotope, and fluorescence showed a pooled metastatic rate of 17.8% (95% CI 14.4%-21.8%), 12.0% (95% CI 8.2%-17.3%), and 28.6% (95% CI 16.2%-45.4%), respectively. The Asian studies showed a slightly higher metastatic rate than the North American studies as well as European studies. We only detected statistically significant heterogeneity in the subgroup of Asian studies. The detailed data were summarized in Table 3. The coefficient was not statistically significant for sample size in further meta-regression analysis (P = 0.17).

**Fig 5:**
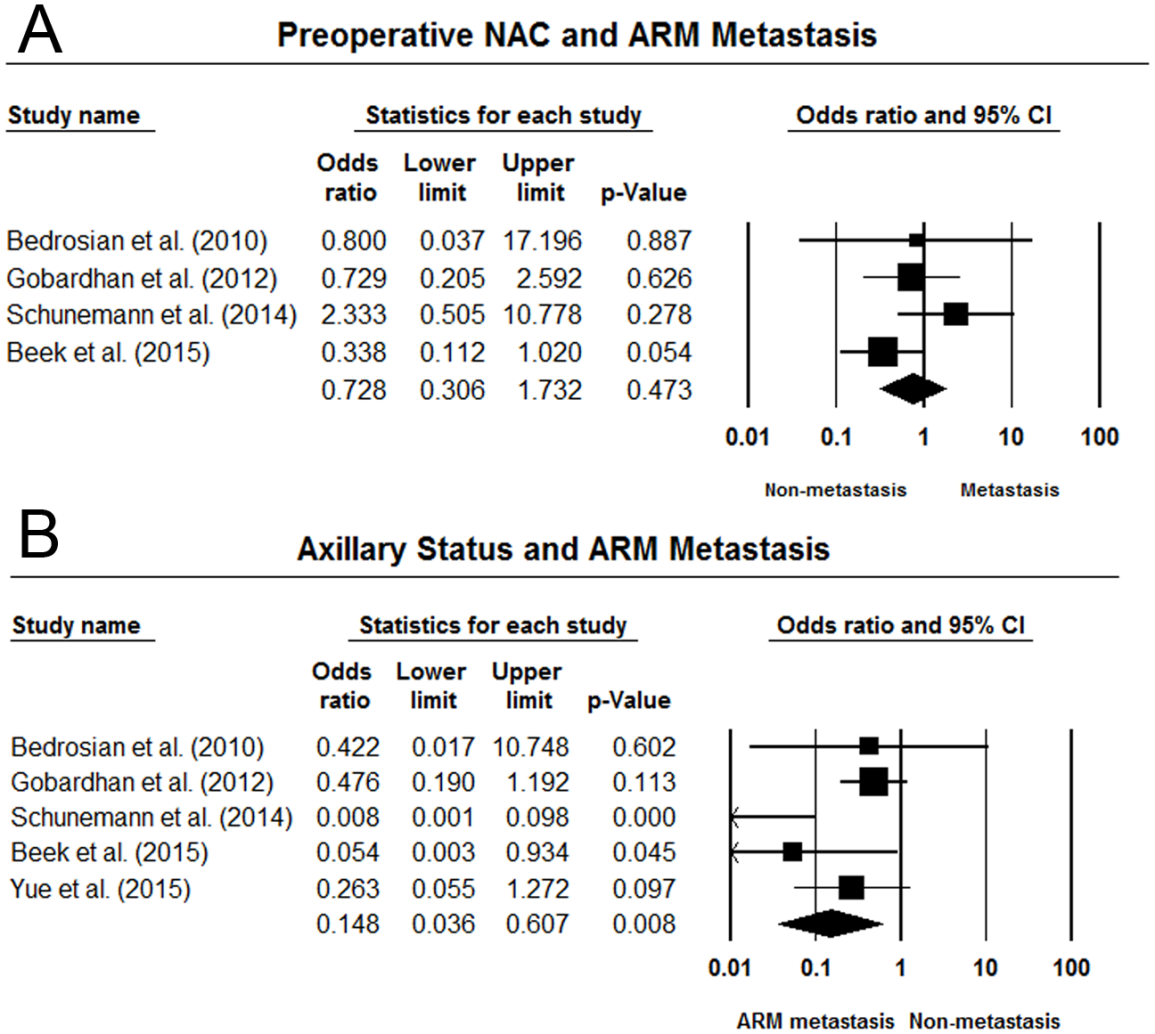
Forest plots of the association between preoperative neoadjuvant chemotherapy, axillary status and the risk of ARM metastasis. (A) preoperative neoadjuvant chemotherapy; (B) axillary status.

Four studies additionally investigated the association between preoperative neoadjuvant chemotherapy (NAC) and metastatic ARM nodes. Nevertheless, the pooled results did not show statistically significant correlation between NAC and ARM-node metastasis (OR = 0.73, 95% CI 0.31–1.73), with low heterogeneity (I^2^ = 26.0%, P = 0.26). (Fig 5A) Five ALND studies investigated the impact of clinical stages on the metastatic rate of ARM nodes [26, 29, 40–42]. We compared the metastatic rates between pN_0-1_ and pN_2-3_ groups. The pooled data indicated that patients of stage pN_0-1_ showed significantly increased risk of ARM metastasis compared with patients of stage pN_2-3_ (OR = 0.15, 95% CI 0.04–0.61, P < 0.05), with statistically significant heterogeneity (I^2^ = 61.2%, P < 0.05). (Fig 5B)

### Lymphedema

**Fig 6:**
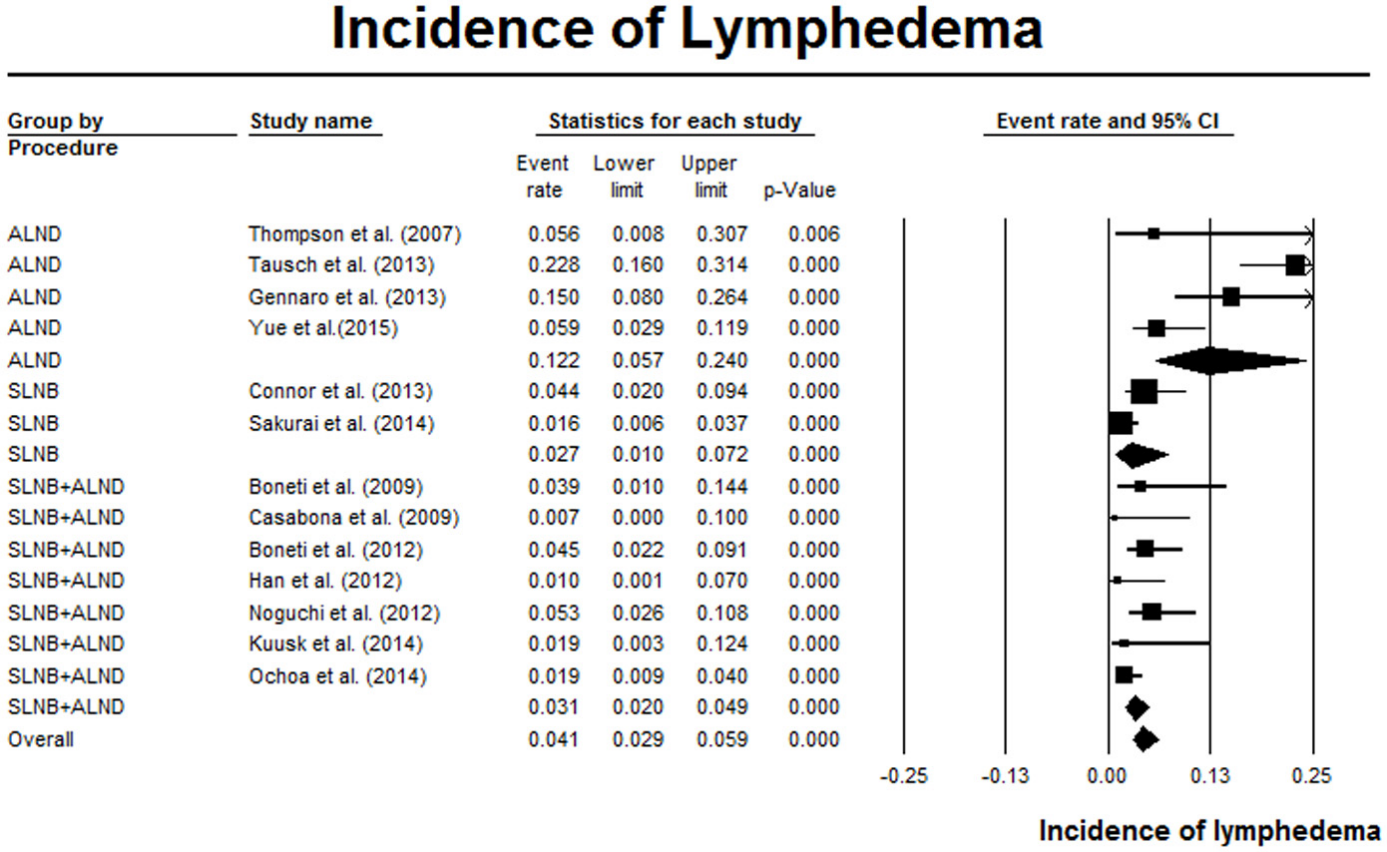
Forest plot of the pooled incidence of lymphedema, which was stratified by different procedures.

Thirteen studies reported the incidence of lymphedema during follow-up [5, 7, 9, 11, 28, 30, 31, 33, 34, 37–39, 42]. The follow-up duration ranged from 6 months to 24 months (Table 1). The overall lymphedema incidence was 4.1% (95% CI 2.9–5.9%), with statistically significant heterogeneity (I^2^ = 85%, P < 0.05). In subgroup analyses, studies of ARM during ALND alone showed much higher incidence of lymphedema (12.2%, 95% CI 5.7–24%, I^2^ = 77.4%, P < 0.05) than SLNB alone (2.7%, 95% CI 1.0%-7.2%, I^2^ = 66.6%, P = 0.08) or the mixed group (3.1%, 95% CI 2.0%-4.9%, I^2^ = 19.0%, P = 0.29). (Fig 6) Only Yue et al. conducted a RCT to compare the incidence of lymphedema between ARM group and non-ARM group, showing that non-ARM patients had a higher incidence when compared with the ARM patients (33.1% versus 5.9%, P < 0.001) [42]. However, meta-analysis for comparison was not performed due to insufficient data.

## Discussion

The results of our meta-analysis demonstrated that the pooled identification rate of ARM lymphatics or nodes was 82.8% during ALND, which was much higher than that during SLNB (38.2%). This discrepancy was supported by the suggestion that the majority of lymphatics draining the upper extremity may be located deeper than the SLNs [9]. Most studies used blue dye alone as mapping material for ARM identification. Compared with blue dye alone, the fluorescent imaging and combined use of blue dye with radioisotope seemed to be more sensitive for detecting ARM lymphatic systems during ALND procedures. In addition, the detection failure may be attributed to the existence of learning curve, the insufficient time interval elapsing from blue dye injection to initiation of surgery, the potential location of nodes outside the axilla area, or the extensive metastasis of ARM nodes obstructing the lymphatic drainages [6, 25, 34].

The crossover rate of SLN-ARM nodes by using blue dye injection was 7.8%. It would be difficult to preserve the converged SLN-ARM nodes during SLNB. Thus, the ability of ARM to prevent lymphedema may be limited after removing these converged nodes. With respect to the pathologic status of resected ARM nodes, the overall metastatic rate was 16.9%. This may be explained by that the ARM nodes were located in the central nodal group for breast lymphatic drainage, which was also supported by the existence of converged SLN-ARM nodes [43]. Additionally, the numerous interconnections shared by lymphatic drainages of the arm and the breast may contribute to ARM metastasis [20]. Preoperative neoadjuvant chemotherapy did not significantly decrease the risk of ARM-nodes metastasis. However, patients with extensive axillary metastasis carried an increased risk of metastasis to the ARM nodes. Therefore, ARM may be contraindicated for patients with clinically positive breast cancer.

The incidence of lymphedema post ARM procedures was 4.1% during follow-up. Recently, a meta-analysis of 72 studies showed that the pooled incidence of arm lymphedema was 19.9% in ALND, and was 5.6% in SLNB [14]. As only 1 ARM study was included in this meta-analysis [30], the pooled results represented the overall incidence of lymphedema in non-ARM procedures. Therefore, it appeared that ARM was effective in preventing lymphedema. A higher incidence of lymphedema was revealed for ARMs during ALND procedures compared with ARMs during SLNB procedures. This discrepancy may be attributed to that the majority of lymphatics draining the upper extremity were located deep to the plane of SLNs, thus causing more disruptions of the lymphatic during ALND [44]. In one study comparing the ARM-nodes preservation group with the ARM-nodes resection group, patients with preserved ARM nodes experienced significantly decreased incidence of lymphedema [33]. In accordance, several studies demonstrated that lymphedema mostly occurred accompanying with the resection of ARM lymphatic nodes or lymphatics [5, 7, 24, 30, 31, 34, 42].

We were aware of the limitations regarding this meta-analysis. Except for 1 RCT comparing the incidence of lymphedema between ARM and non-ARM procedures [42], most publications were single-arm studies of ARM procedures, which precluded the availability of direct comparison effect estimates. Thus, the efficacy of ARM in preventing lymphedema could not be thoroughly evaluated by controlled groups. We could only try to compare it with previous meta-analysis results. The efficacy outcome did not serve as one of our main objectives. Although meta-analysis of RCTs provided the best evidence, our pooled results from non-randomized studies were of clinical significance to inform the design of subsequent trials that evaluate the long-term efficacy of ARM in preventing lymphedema [45]. Additionally, the clinical features, such as ages, breast cancer stages, and preoperative NAC, were heterogeneous among included studies. For example, several studies clearly excluded patients who had received NAC [5, 9, 27, 31, 42]. Besides, the definition, measurement and follow-up duration of lymphedema were inconsistent across included studies. Some clinical variables may be associated with the risk of lymphedema, such as body mass index and the receipt of radiation therapy or chemotherapy, which were not adjusted or balanced in most studies [14]. Further well-designed RCTs were warranted to provide more convincing evidence.

We noted that a review has described ARM in depth recently [43]. In comparison, the distinct features and strengths of our study lied in the following aspects. Our study represented the first meta-analysis regarding ARM procedures, which included a large number of prospective studies through comprehensive literature search. The rates relating to the feasibility and oncological safety of ARM procedures were statistically summarized, with separate exploration for SLNB and ALND. The impact of NAC and axillary status on the metastasis of ARM nodes were firstly systematically analyzed. Besides, the included studies were critically appraised by quality tool, displaying moderate to high methodological qualities. The heterogeneity was carefully explored by subgroup analyses and meta-regression analyses. No publication bias was detected for included studies.

## Conclusion

The ARM technique was feasible for patients undergoing ALND, but was limited by unsatisfying identification rate of ARM nodes for patients undergoing SLNB. ARM appeared to be beneficial for decreasing the occurrence of arm lymphedema. However, clinicians should prudently perform this procedure in light of the possibility of crossover SLN-ARM nodes or metastatic ARM nodes. Patients with clinically positive breast cancer may be unsuitable for ARM due to potentially increased risk of ARM-nodes metastasis.

## Supporting Information

S1 PRISMA Checklist PRISMA Checklist. (DOC)

S1 PRISMA Diagram PRISMA 2009 Flow Diagram. (DOC)

S1 Table The search strategy of this systematic review and meta-analysis. (DOCX)

S2 Table Results of quality assessment by the Agency for Healthcare Research and Quality (AHRQ) checklist. (DOCX)

S1 Text Full-text excluded articles. (DOCX)
