# Supplementary material for: The Feasibility and Oncological Safety of Axillary Reverse Mapping in Patients with Breast Cancer: A Systematic Review and Meta-Analysis of Prospective Studies
Source: PLoS One. 2016 Feb 26;11(2):e0150285. doi: 10.1371/journal.pone.0150285 (PMC4769133; doi:10.1371/journal.pone.0150285)
Supplement: S1 PRISMA Diagram — (DOC) [file pone.0150285.s002.doc]

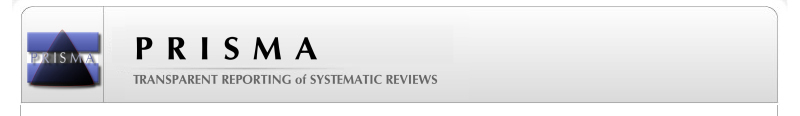
**PRISMA 2009 Flow Diagram**

**Screening**

**Included**

**Eligibility**

**Identification**

Full-text articles assessed for eligibility
(n = 32)

Studies included in qualitative synthesis
(n = 28)

Studies included in quantitative synthesis (meta-analysis)
(n = 24)

Records identified through database searching:

Medline (43)

Embase (53)

Cochrane Library (2)

Web of Science (51)

Scopus (55)

(n = 204)

Duplicates removed (n = 142)

**Excluded for the following:**-Review or consensus (n = 11)

-Editorials or letter to the editor (n = 9)

- Irrelevant study ( n = 3)

-Only abstract (n = 7)

**Excluded for the following**:

-Studies with insufficient data (n = 1)

-Case report (n = 1)

-Postmortem study (n = 1)

-Trial protocol (n = 1)

**Excluded for the following**:

-Retrospective study (n = 1)

-Studies of overlapping population (n = 3)

Studies of duplicated cohort (n = 1)

Records screened

(n = 62)
