# Supplementary material for: The Feasibility and Oncological Safety of Axillary Reverse Mapping in Patients with Breast Cancer: A Systematic Review and Meta-Analysis of Prospective Studies
Source: PLoS One. 2016 Feb 26;11(2):e0150285. doi: 10.1371/journal.pone.0150285 (PMC4769133; doi:10.1371/journal.pone.0150285)
Supplement: S1 Table — (DOCX) [file pone.0150285.s003.docx]

**S1 Table. Search strategy of this systematic review and meta-anaysis**

| Data source | Search strategy |
| --- | --- |
| Pubmed | #1 breast neoplasm [MeSH Terms]  #2 breast cancer* [Text Word]) OR breast carcinoma [Text Word]) OR breast neoplasm* [Text Word]) OR breast tumor* [Text Word]) OR breast tumour* [Text Word])  #3 breast cancer* OR breast carcinoma OR breast neoplasm* OR breast tumor* OR breast tumour*  #4 ((#1) OR #2) OR #3  #5 axillary reverse mapping [Text Word] OR lymphatic arm drainage [Text Word] OR axillary reverse lymphatic mapping [Text Word]  #6 axillary reverse mapping [Title/Abstract] OR lymphatic arm drainage [Title/Abstract] OR reverse lymphatic mapping [Title/Abstract]  #7 #5 OR #6  #8 #7 AND #4  #9 #8 AND English[Language] |
| Embase | #1 'breast cancer'/exp OR 'breast cancer'  #2 breast:ab,ti OR mammary:ab,ti OR mammaries:ab,ti AND (cancer*:ab,ti OR tumour*:ab,ti OR tumor*:ab,ti OR neoplasm*:ab,ti OR metastas*:ab,ti OR carcinoma*:ab,ti)  #3 #1 OR #2  #4 'axillary reverse mapping'  #5 'lymphatic arm drainage'  #6 'reverse lymphatic mapping'  #7 #4 OR #5 OR #6  #8 #2 AND #7 AND [humans]/lim AND [english]/lim AND ([article]/lim OR [article in press]/lim OR [editorial]/lim OR [erratum]/lim OR [letter]/lim OR [note]/lim OR [review]/lim OR [short survey]/lim) |
| Cochrane Library | #1“breast cancer*” OR “breast carcinoma” OR “breast neoplasm*” OR “breast tumor*” OR “breast tumour*”  #2 "axillary reverse mapping" OR "lymphatic arm drainage" OR "reverse lymphatic mapping"  #3 #1 AND #2 |
| Web of Science | #1 TS=("breast cancer*") OR TS=("breast carcinoma") OR TS=("breast neoplasm*") OR TS=("breast tumor*") OR TS=("breast tumour*")  #2 TS=("axillary reverse mapping") OR TS=("lymphatic arm drainage") OR TS=("axillary reverse lymphatic mapping")  #3 #1 AND #2 |
| Scopus | #1 TITLE-ABS-KEY ("breast cancer*" OR "breast carcinoma" OR "breast neoplasm*" OR "breast tumor*" OR "breast tumour*")  #2 TITLE-ABS-KEY ("axillary reverse mapping" OR "lymphatic arm drainage" OR "reverse lymphatic mapping")  #3 ( TITLE-ABS-KEY ("breast cancer*" OR "breast carcinoma" OR "breast neoplasm*" OR "breast tumor*" OR "breast tumour*") ) AND ( TITLE-ABS-KEY ("axillary reverse mapping" OR "lymphatic arm drainage" OR "reverse lymphatic mapping") )  #4 ( TITLE-ABS-KEY ("breast cancer*" OR "breast carcinoma" OR "breast neoplasm*" OR "breast tumor*" OR "breast tumour*") ) AND ( TITLE-ABS-KEY ("axillary reverse mapping" OR "lymphatic arm drainage" OR "reverse lymphatic mapping") ) AND LANGUAGE (english ) |
