# Supplementary material for: The Feasibility and Oncological Safety of Axillary Reverse Mapping in Patients with Breast Cancer: A Systematic Review and Meta-Analysis of Prospective Studies
Source: PLoS One. 2016 Feb 26;11(2):e0150285. doi: 10.1371/journal.pone.0150285 (PMC4769133; doi:10.1371/journal.pone.0150285)
Supplement: S2 Table — (DOCX) [file pone.0150285.s004.docx]

**S2 Table. Quality assessment of included studies by using the Agency for Healthcare Research and Quality (AHRQ) checklist.**

|  | Thompson et al. (2007) | Nos et al. (2007) | Nos et al. (2008) | Boneti et al. (2009) | Casabona et al. (2009) | Ponzone et al. (2009) | Bedrosian et al. (2010) | Deng et al. (2011) | Boneti et al. (2012) | Gobardhan et al. (2012) | Han et al. (2012) | Rubio et al. (2012) | Noguchi et al. (2012) | Connor et al. (2013) | Tausch et al. (2013) | Gennaro et al. (2013) | Ikeda et al. (2014) | Khandelwal et al. (2014) | Kuusk et al. (2014) | Ochoa et al. (2014) | Sakurai et al. (2014) | Schunemann et al. (2014) | Beek et al. (2015) | Yue et al. (2015) |
| --- | --- | --- | --- | --- | --- | --- | --- | --- | --- | --- | --- | --- | --- | --- | --- | --- | --- | --- | --- | --- | --- | --- | --- | --- |
| Define the source of information (surgery, record review) | Y | Y | Y | Y | Y | Y | Y | Y | Y | Y | Y | Y | Y | Y | Y | Y | Y | Y | Y | Y | Y | Y | Y | Y |
| List inclusion and exclusion criteria | N | N | N | Y | Y | Y | Y | Y | N | N | Y | N | Y | Y | N | N | Y | Y | Y | N | Y | Y | N | Y |
| Indicate time period used for identifying patients | Y | Y | Y | Y | Y | Y | Y | Y | Y | Y | Y | Y | Y | Y | Y | Y | Y | Y | Y | Y | Y | Y | Y | Y |
| Indicate whether or not subjects were consecutive if not population-based | Y | U | U | U | U | Y | U | U | U | U | U | U | U | U | U | U | U | U | U | U | U | U | Y | U |
| Indicate if evaluators of subjective components of study were masked to other aspects of the status of the participants | N | N | N | N | N | N | N | N | N | N | N | N | N | N | N | N | N | N | N | N | N | N | N | Y |
| Describe any assessments undertaken for quality assurance purposes | Y | Y | Y | Y | Y | Y | Y | Y | Y | Y | Y | Y | Y | Y | Y | Y | Y | Y | Y | Y | Y | Y | Y | Y |
| Explain any patient exclusions from analysis | N | U | U | U | U | U | U | U | U | U | U | U | U | U | Y | U | Y | U | U | U | Y | U | U | U |
| Describe how confounding was assessed and/or controlled | N | N | N | Y | Y | Y | Y | Y | U | Y | N | N | N | Y | Y | N | Y | N | N | N | N | Y | Y | Y |
| If applicable, explain how missing data were handled in the analysis | U | U | U | U | U | U | U | U | U | U | U | U | U | U | U | U | U | U | U | U | U | U | U | U |
| Summarize patient response rates and completeness of data collection | U | U | U | U | U | U | U | U | U | U | U | U | U | U | U | U | U | U | U | U | U | U | U | U |
| Clarify what follow-up, if any, was expected and the percentage of patients for which incomplete data or follow-up was obtained | Y | Y | N | Y | N | Y | N | N | Y | N | N | Y | Y | N | Y | Y | N | Y | Y | Y | Y | N | N | Y |
| Total items (NA items were excluded from the sum) | 4 | 4 | 2 | 6 | 4 | 7 | 4 | 4 | 4 | 3 | 3 | 4 | 5 | 4 | 6 | 4 | 5 | 5 | 5 | 4 | 6 | 4 | 4 | 7 |

Y, yes; N, no; U, unclear.
