# Supplementary material for: The Feasibility and Oncological Safety of Axillary Reverse Mapping in Patients with Breast Cancer: A Systematic Review and Meta-Analysis of Prospective Studies
Source: PLoS One. 2016 Feb 26;11(2):e0150285. doi: 10.1371/journal.pone.0150285 (PMC4769133; doi:10.1371/journal.pone.0150285)
Supplement: S1 Text — (DOCX) [file pone.0150285.s005.docx]

**Three studies had overlapping patient population with the included studies**

1. Boneti C, Korourian S, Bland K, Cox K, Adkins LL, Henry-Tillman RS, et al. Axillary reverse mapping: mapping and preserving arm lymphatics may be important in preventing lymphedema during sentinel lymph node biopsy. Journal of the American College of Surgeons. 2008;206(5):1038-42; discussion 42-4. doi: 10.1016/j.jamcollsurg.2007.12.022. PubMed PMID: 18471751.

2. Ikeda K, Ogawa Y, Komatsu H, Mori Y, Ishikawa A, Nakajima T, et al. Evaluation of the metastatic status of lymph nodes identified using axillary reverse mapping in breast cancer patients. World journal of surgical oncology. 2012;10:233. doi: 10.1186/1477-7819-10-233. PubMed PMID: 23116152; PubMed Central PMCID: PMC3527301.

3. Noguchi M, Yokoi M, Nakano Y. Axillary reverse mapping with indocyanine fluorescence imaging in patients with breast cancer. Journal of surgical oncology. 2010;101(3):217-21. doi: 10.1002/jso.21473. PubMed PMID: 20063370.

**One case report**

Gandhi SJ, Satish C, Shanmuga Sundaram P, Subramanyam P, Vijaykumar DK. Axillary reverse mapping using 99mTc-SC: a case illustration. Clinical nuclear medicine. 2014;39(10):e428-30. doi: 10.1097/RLU.0000000000000256. PubMed PMID: 24152646.

**One trial protocol**

Klompenhouwer EG, Gobardhan PD, Beek MA, Voogd AC, Luiten EJ. The clinical relevance of axillary reverse mapping (ARM): study protocol for a randomized controlled trial. Trials. 2013;14:111. doi: 10.1186/1745-6215-14-111. PubMed PMID: 23782712; PubMed Central PMCID: PMC3663653.

**One study of insufficient data**

1. Pavlista D, Eliska O. Analysis of direct oil contrast lymphography of upper limb lymphatics traversing the axilla -- a lesson from the past -- contribution to the concept of axillary reverse mapping. European journal of surgical oncology : the journal of the European Society of Surgical Oncology and the British Association of Surgical Oncology. 2012;38(5):390-4. doi: 10.1016/j.ejso.2012.01.010. PubMed PMID: 22336143.

**One retrospective study**

Pasko JL, Garreau J, Carl A, Ansteth M, Glissmeyer M, Johnson N. Axillary reverse lymphatic mapping reduces patient perceived incidence of lymphedema after axillary dissection in breast cancer. American journal of surgery. 2015;209(5):890-5. doi: 10.1016/j.amjsurg.2015.01.011. PubMed PMID: 25796096.

**One postmortem study**

Pavlista D, Eliska O. Relationship between the lymphatic drainage of the breast and the upper extremity: a postmortem study. Annals of surgical oncology. 2012;19(11):3410-5. Epub 2012/04/25. doi: 10.1245/s10434-012-2363-x. PubMed PMID: 22526910.
